# Prevalence of Isthmi and Root Canal Configurations in Mandibular Permanent Teeth Using Cone-Beam Computed Tomography

**DOI:** 10.1155/2024/9969860

**Published:** 2024-07-31

**Authors:** Sherwin Arman, Ahmad Nouroloyouni, Amin Salem Milani, Behzad Sheikhfaal, Sara Noorolouny, Faraz Saleh Haghgou, Hesam Mikaieli Xiavi

**Affiliations:** ^1^ Orofacial Pain Program Section of Oral Medicine Oral Pathology and Orofacial Pain University of California Los Angeles School of Dentistry, Los Angeles, California, USA; ^2^ Department of Endodontics School of Dentistry Ardabil University of Medical Science, Ardabil Po. Code 5618985991, Iran; ^3^ Department of Endodontics School of Dentistry Tabriz University of Medical Science, Tabriz Po. Code 5165665931, Iran; ^4^ Department of Pediatric Dentistry School of Dentistry Ardabil University of Medical Science, Ardabil Po. Code 5618985991, Iran; ^5^ Department of Radiology Ardabil University of Medical Science, Ardabil Po. Code 5618985991, Iran

**Keywords:** cone-beam computed tomography, endodontics, mandible, Middle East, tooth root

## Abstract

While root canal anatomy in Middle Eastern populations is well-studied, research on isthmi in mandibular permanent teeth from this region is limited. This retrospective study used cone-beam computed tomography (CBCT) to examine isthmi prevalence and location, as well as root canal morphologies (per Vertucci's classification) in mandibular permanent teeth from a subpopulation in Ardabil, Iran. The study is aimed at enhancing our understanding of dental anatomical variations in Middle Eastern populations. A total of 3566 teeth from 384 CBCT scans were evaluated in this retrospective study. Mandibular teeth were evaluated on sagittal, coronal, and axial sections regarding the presence of isthmus and root morphology (Vertucci's classification). CBCT scans of 197 males (51.3%) and 187 females (48.7%) were evaluated, with a mean age of 41.1 ± 11.4 years. Isthmi were most prevalent in molar mesial roots, typically located in the middle third for anterior teeth and first premolars and cervical third for posterior teeth. While gender did not play a significant role, the presence of an isthmus in the mesial root of the left second molars was associated with a younger mean patient age (*p* < 0.05). Root morphology varied across tooth types. Central and lateral incisors predominantly showed Vertucci's Types I and III. Canines and premolars were mostly Type I, with some variation. Molar mesial roots frequently exhibited Types IV and II, while distal roots were predominantly Type I. Statistically significant differences were found between morphology and gender in the first left premolar (Type I more common in women; Type V in men; *p* < 0.001) and in the right canine (Type I more prevalent in men; Types III and V more prevalent in women; *p* < 0.001). The results revealed wide variations in root canal morphology and a relatively high prevalence of isthmi in the study population. Our findings suggest a potential difference in tooth anatomy based on sex and a relationship between age and the presence of isthmi.

## 1. Introduction

Comprehensive understanding of dental anatomy and the intricate root canal system is paramount for successful endodontic treatment [1]. Root canal therapy is aimed at achieving complete debridement, eliminating microorganisms and their byproducts from the root canal system [2]. The original morphology significantly impacts treatment quality, and unfamiliarity with the diversity of root canal anatomies is a major contributor to endodontic failures [3, 4].

An isthmus refers to a thin, elongated area within the pulp space that connects or links different parts of the root canal system. It can appear as a slender extension, a side connection, or a cross-linking passage between one or two root canals [5, 6]. Isthmi have been reported to have a relatively high prevalence, underscoring the importance of adequate disinfection in these regions during root canal therapy [7, 8].

The presence of isthmi in root canal systems can harbor necrotic material, remnants of tissue, or organic matter, potentially fostering microbial growth and compromising the success of endodontic procedures [1, 6]. There is not sufficient data available in the literature to fully comprehend how the preparation of isthmi affects the effectiveness of nonsurgical endodontic treatments. To address this knowledge gap, it is necessary to conduct studies that track patient outcomes over extended periods [9].

In our studied population, missed canals were among the most frequent iatrogenic errors in root canal treatment of mandibular teeth [10, 11]. Up to 40% of distolingual canals were overlooked in mandibular molars in one report from our studied population [11]. A primary etiological factor contributing to the missed canal phenomenon is the clinician's incomplete comprehension of the intricate and variable nature of root canal system anatomy, as well as racial factors influencing the prevalence of anatomical features [12, 13]. Additional compounding variables include inadequate magnification and illumination, the presence of calcifications obscuring canal orifices, and the inherent complexity of the root canal system configuration [12].

However, significant advancements in endodontists' understanding of root canal anatomy have occurred over the past two decades, facilitated by the incorporation of surgical operating microscopes and cone-beam computed tomographic (CBCT) imaging modalities into contemporary clinical practice. These technological adjuncts have provided enhanced visualization and three-dimensional representations of the root canal system, enabling more accurate identification and management of anatomical complexities during root canal treatment [12]. CBCT provides images of different sections of an object in axial, sagittal, and coronal planes. By 3D reconstruction of images, CBCT can aid in the detailed assessment of the anatomy and morphology of structures. CBCT is noninvasive, and the specimens remain intact after assessment, which is an advantage for in vitro studies [14, 15].

Evidence shows that race and ethnicity play important roles in the prevalence of isthmi and anatomical variations of teeth and the root canal system. Thus, studies on different populations and racial and ethnic groups are required to obtain more precise data regarding root canal morphology and anatomical variations in different populations [16, 17]. While root canal anatomy in Middle Eastern populations is well-studied, research on isthmi in mandibular permanent teeth from this region is limited. This data seems necessary to perform a successful endodontic treatment as well as for target-based continuing education based on the needs and information of each area [10, 11]. This study is aimed at assessing the root canal morphology and prevalence of isthmi and their location (if present) in mandibular teeth in Ardabil city, Iran. The null hypothesis is that there is no significant difference in the prevalence of isthmi and root canal configurations in mandibular permanent teeth among different tooth types, age groups, or between genders.

## 2. Materials and Methods

### 2.1. Ethical Consideration

The study was approved by the ethics committee of Ardabil University of Medical Sciences (IR.ARUMS.REC.139). Patients consented to the use of their CBCT scans for research purposes at the time of imaging.

### 2.2. Data Collection Procedure

This retrospective study was performed on CBCT scans retrieved from archives of the only two radiology clinics in Ardabil city. The CBCT scans had been taken for purposes not related to this study such as tooth extraction or orthodontic treatment in a period of 1 year. All patients gave their written informed consent for the possible use of their CBCT scans for research purposes. The sample size was estimated to be 384 according to Morgan's table, as there were 2240 CBCTs available. The first 384 scans meeting the following criteria were selected.

The inclusion criteria were high-quality CBCT scans of mandibular teeth with completely formed roots. Third molars, teeth with developmental anomalies, endodontically treated teeth, teeth under prosthetic crowns and deep restorations (due to artifact), teeth under orthodontic treatment, and pathological lesions, or internal/external root resorption were excluded. All CBCT scans were obtained using the NewTom GiANO (Verona, Italy) CBCT scanner with settings of 90 kVp, 3 mA, voxel size of 0.2 mm, a field of view of 11 cm by 13 cm, and an exposure time of 9 s. The scans were performed by an oral and maxillofacial radiologist. NNT software (NewTom 5G, QR, Verona, Italy) was used to evaluate the images. If necessary, the image contrast and brightness were adjusted to achieve optimal visualization.

**Figure 1:**
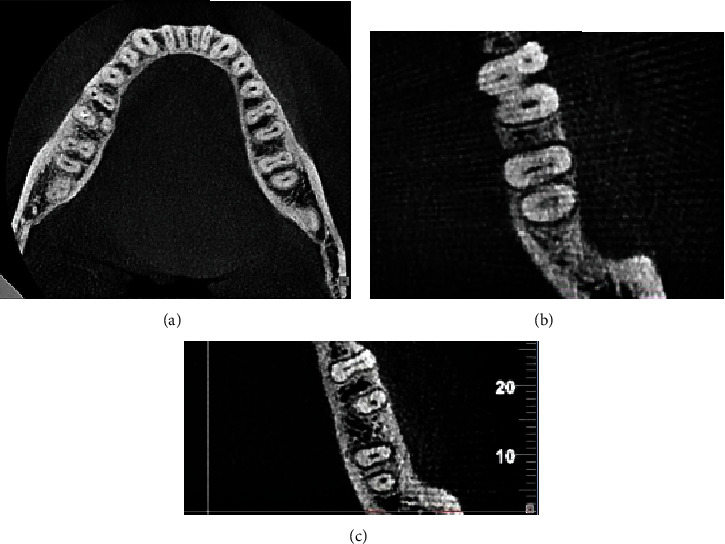
Different locations of isthmi in axial view: (a) Isthmus is present in the mesial and distal roots of the right first molar and mesial root of the second molar. (b) Isthmus is present only in the mesial root of the mandibular second molar. (c) Isthmus is present only in the mesial root of the mandibular first molar.

**Figure 2:**
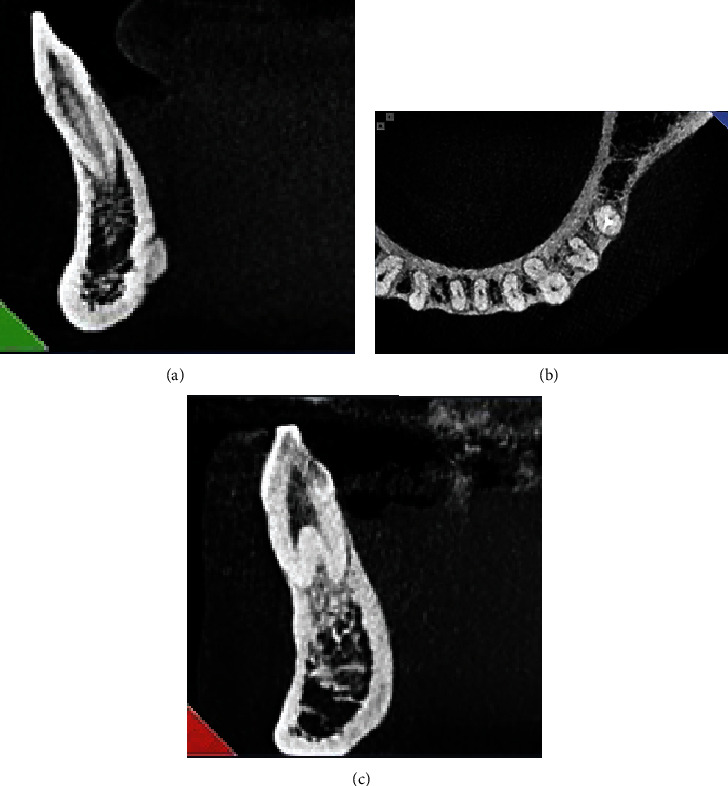
(a) Sagittal, (b) axial, and (c) coronal views of the CBCT image to evaluate the configuration of the root canal.

On eligible CBCT scans, the teeth were evaluated regarding the presence of isthmi (Figure 1). Root canal morphology was also evaluated by a calibrated dental student under the supervision of an endodontist and oral and maxillofacial radiologist and classified according to Vertucci's classification (Figure 2) [13]. The age, gender, right or left sides, location of the isthmus (if present), and type of root canal morphology according to Vertucci's classification were recorded. To assess the intraobserver reliability, 25% of the images were assessed again after a 2-week period. The level of agreement between the two observations was found to be kappa = 0.9 indicating excellent agreement was recorded for each CBCT scan.

In order to compare the findings of this study with similar ones and because the Vertucci classification system is the standard method for classifying root canal configurations, this system was used in this study. Vertucci classified root canal configurations into eight types [18]:

Type I: A single canal extends from the pulp chamber to the apex.

Type II: Two separate canals leave the pulp chamber and join short of the apex to form one canal.

Type III: One canal leaves the pulp chamber, divides into two within the root, and then merges to exit as one canal.

Type IV: Two separate and distinct canals extend from the pulp chamber to the apex.

Type V: One canal leaves the pulp chamber and divides short of the apex into two separate and distinct canals with separate apical foramina.

Type VI: Two separate canals leave the pulp chamber, merge in the body of the root, and redivide short of the apex to exit as two distinct canals.

Type VII: One canal leaves the pulp chamber, divides and then rejoins within the body of the root, and finally, redivides into two distinct canals short of the apex.

Type VIII: Three separate and distinct canals extend from the pulp chamber to the apex.

The location of isthmi was categorized as follows [19]:

CT-CT: initiation and termination in the cervical third.

CT-MT: initiation in the cervical third and termination in the middle third.

CT-AT: initiation in the cervical third and termination in the apical third.

MT-MT: initiation and termination in the middle third.

MT-AT: initiation in the middle third and termination in the apical third.

AT-AT: initiation and termination in the apical third.

Data were reported as mean and standard deviation for quantitative variables, and frequency and percentage for qualitative variables. The collected data were analyzed by SPSS 25. The correlation of age with the prevalence of isthmi was analyzed by an independent *t*-test, while the correlation of age with root canal morphology was evaluated by a one-way analysis of variance (ANOVA). The Monte Carlo test was also used to examine the statistically significant relationship between the frequency of root canal morphology types in the right or left side teeth and the patient's gender. The level of significance was set at 0.05.

## 3. Results

Of 384 patients, 197 (51.3%) were males, and 187 (48.7%) were females. The mean age of the participants was 41.1 ± 11.4 years (range 10 to 72 years).

### 3.1. Root Canal Morphology According to Vertucci's Classification and Their Relationship With Age and Gender

**Figure 3:**
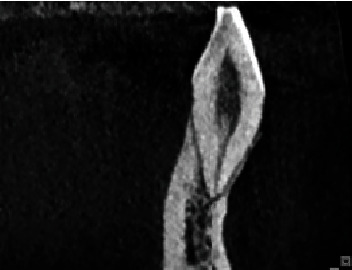
Sagittal view showing Vertucci Type III canal configuration in mandibular central and lateral incisors, which is the second most frequent anatomical configuration.

**Figure 4:**
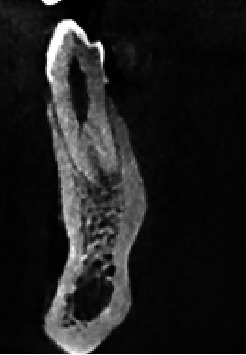
Coronal view showing Vertucci Type V canal configuration in mandibular first premolar, which is the second most frequent anatomical configuration.

Tables 1 and 2 show the frequency of root canal morphology of mandibular teeth on the right and left sides, respectively, according to Vertucci's classification. As shown, the most common root morphology was Type I (50.5%–51%) followed by Type III (41.8%–43%) in central incisors, Type I (48.2%–48%) and Type III (45.2%–46.1%) in lateral incisors (Figure 3), Type I (85.5%–87.1%) and Type III (8.2%–9.4%) in canine teeth, Type I (72.6%–77.2%) and Type V (19%–19.9%) (Figure 4) in first premolars, Type I (91.6%–93.8%) in second premolars, Type IV (58.3%–64%) and Type II (30.7%–34.7%) in mesial root of first molars, Type I (69.4%–73.3%) and Type II (11.1%–12%) in distal root of first molars, Type IV (43.1%–46.3%) and Type II (31.7%–36.2%) in mesial root of second molars, and Type I (91.6%–92.7%) in distal root of second molars, in the right and left sides, respectively.

The Monte Carlo test was used to examine the statistically significant relationship between the frequency of root canal morphology types in the left teeth and the patient's gender. The significance level of the test was 0.05. The test results (presented in Table 1) showed the following:

1. There was a statistically significant relationship between the frequency of root canal morphology types in the left first premolar tooth and the patient's gender. The frequency of Type I morphology was 141 in females and 52 in males, while the frequency of Type V (Figure 4) morphology was 9 in females and 44 in males. This difference was statistically significant (*P* value < 0.001).
2. There was no statistically significant relationship between the frequency of root canal morphology types in the other left teeth under study and the patient's gender (*P* value > 0.05).

The Monte Carlo test was also used to examine the statistically significant relationship between the frequency of root canal morphology types in the right teeth and the patient's gender. The significance level of the test was 0.05. The test results (presented in Table 2) showed the following:

1. There was a statistically significant relationship between the frequency of root canal morphology types in the right canine tooth and the patient's gender. The frequency of Type I morphology was 182 in males and 119 in females, the frequency of Type III morphology was 11 in males and 22 in females, and the frequency of Type V morphology (Figure 2(c)) was 3 in males and 13 in females. This difference was statistically significant (*P* value < 0.001).
2. There was no statistically significant relationship between the frequency of root canal morphology types in the other right teeth under study and the patient's gender (*P* value > 0.05).

To investigate the presence of a statistically significant difference between the mean age of study participants based on root canal morphology, a one-way ANOVA was used. The results of this test showed that for none of the teeth under study, there was a statistically significant difference between the mean age of individuals based on root canal morphology.

### 3.2. Prevalence of Isthmi in the Root Canals of Mandibular Teeth

Table 3 presents the frequency of isthmi in the root canals of mandibular teeth on the right and left sides. The current results showed the highest frequency of isthmi in the mesial root of the first and second molars (Figure 1), followed by the distal root of the first molars, lateral incisors, and central incisors. The prevalence of isthmi was low in the remaining teeth.

### 3.3. Prevalence of Isthmi in the Root Canals of Mandibular Teeth Based on Age

To investigate the normality of the age variable distribution, the Kolmogorov–Smirnov test was used. The significance level of the test was 0.05. The results of this test showed that the age variable distribution was normal (*P* value > 0.05).

To investigate the presence of a statistically significant difference between the mean age of study participants based on the presence or absence of an isthmus, the independent sample *T*-test was used. The results of this test, presented in Table 4, showed the following:

In the mesial root of the second left molar (7LM), there was a statistically significant difference between the mean age of individuals based on the presence or absence of an isthmus in the root. The mean age of individuals who had an isthmus in the root of the mentioned tooth was lower than the mean age of individuals who did not have an isthmus in the root of the mentioned tooth (Figures 1(b) and 1(c)) (*P* value < 0.05).

There was no other statistically significant difference between the mean age of individuals based on the presence or absence of an isthmus in the root (*P* value > 0.05).

To investigate the presence of a statistically significant difference between the mean age of study participants based on the presence or absence of an isthmus on the left side, an independent sample *T*-test was also used. The results of this test, presented in Table 5, showed that for none of the teeth under study, there was a statistically significant difference between the mean age of individuals based on the presence or absence of an isthmus in the root (*P* value > 0.05).

### 3.4. Location of Isthmi

Tables 6 and 7 show the frequency distribution of the location of isthmi in the root canals of right and left mandibular teeth. As indicated, isthmi were mainly in the middle third of the root in central and lateral incisors, canines, and first premolars. They were mainly in the cervical third in the second premolars and the mesial root of the first and second molars.

## 4. Discussion

The current study is unique because it evaluates the prevalence and location of isthmi in all permanent mandibular teeth in a Middle Eastern population and extensively evaluates the root canal configuration of all mandibular permanent teeth, analyzing them based on side, age, and gender. In the studied population, the prevalence of iatrogenic errors and missed and C-shaped canals has previously been evaluated and found to be high, suggesting a probable different prevalence of anatomical morphology in this population [11, 12, 20]. Insufficient data about anatomical features results in inadequate root canal cleaning, shaping, or poor-quality obturation of root canals. Persistent endodontic infection can be attributed to missed canals and difficulties in removing a bacterial biofilm from root canal ramifications, including isthmi [21]. This study assessed the prevalence and location of isthmi (if present) in mandibular teeth and the prevalence of different root canal morphologies (according to Vertucci's classification) in mandibular teeth in Ardabil city, Iran.

Regarding the presence of isthmi, the current results revealed the highest frequency of isthmi in the mesial root of the second (63.8%) and first molars (60.5%), followed by the distal root of first molars, lateral incisors, and central incisors. Hu et al. [22], in their study, conducted on a Chinese population, and Tahmasbi et al. [23], in their study, in the USA reported the similar results. However, Haghanifar et al. [19] reported much lower prevalence in their study. They reported that the highest prevalence of isthmi in mandibular teeth was in the mesial roots of the mandibular first molars (36%), followed by the mesial root of the second molars (34%). This lower prevalence could be due to the examination of the only continuous connection between two canals in the same root (complete isthmi) in their study. On the other hand, the comparative incidence of isthmi in the other mandibular teeth of this study was similar to the present study. Srivastava et al. [24] reported the prevalence of isthmi in the mesial root of mandibular first molars in Saudi Arabia to be 78.4% on CBCT scans, which was higher than the rate obtained in the present study. Shakeri et al. [25] in Sari, Iran, found that the prevalence of isthmi was 81.2% (males) and 78.9% (females) in the first molars and 78.7% (males) and 70.7% (females) in the second molars. Both values were higher than the corresponding values reported in the present study. The reported variations in the results of studies can be attributed to differences in definitions of variables such as isthmus, as well as differences in sample size, age, gender, race, and ethnicity of participants in different populations.

Regarding the location of isthmi, it should be noted that cleaning and shaping of isthmi that start in the cervical third are relatively easier compared with those starting from the middle or apical third. In the present study, isthmi in the root canals of central and lateral incisors, canines, and the first premolars were mainly in the middle third (MT-MT; i.e., starting and ending in the middle third); whereas isthmi in the root canals of the second premolars and mesial root of the first and second molars were mainly in the cervical third (CT-CT; i.e., starting and ending in the cervical third). Haghanifar et al. [19], in their study, in Babol city, Iran, reported that the majority of isthmi in central and lateral incisors, canines, and the first premolars of the mandible were in the middle third, which was in accordance with the present findings. However, their results regarding the location of isthmi in the roots of other teeth were different from the present findings; they reported a higher prevalence in the apical third. Hu et al. [22] evaluated the location of isthmi in the mesial root of mandibular first molars in a Chinese population and reported that around 30% of isthmi were entirely in the cervical third, 30% were started in the cervical third and ended in the apical third, and 30% were entirely in the apical third. Srivastava et al. [24] evaluated the location of isthmi in the mesial root of mandibular first molars in Saudi Arabia and reported the maximum number of isthmi entirely in the apical third (34%). In 25% of the cases, isthmi initiated from the cervical third and ended in the apical third, while they were entirely in the cervical third in 20% of the cases. Pécora et al. [26], in their study on a Brazilian population, showed that most isthmi were in the apical third in the root canals of the first and second molars. A wide variety of isthmus locations in mandibular molars across different regions emphasizes the importance of the effect of ethnicity on it and the necessity for comprehensive study.

In the present study, no significant gender difference was found regarding the prevalence and location of isthmi in mandibular teeth, aligning with the findings of Srivastava, Alrogaibah, and Aljarbou [24] in Saudi Arabia. While Shakeri et al. [25] reported similar results for first molar mesial roots in Iran, they found a higher prevalence of isthmi in the second molar mesial roots in males. On the other hand, Haghanifar et al. [19] reported a higher prevalence of isthmi in canines in females in Babol, Iran, contrasting the current results. However, they found no gender difference in other teeth.

Given the relatively high prevalence of isthmi in our study population, greater attention should be given to root canal irrigation during orthograde root canal therapy. This would increase the likelihood of irrigants reaching the isthmi, especially in the mesial roots of mandibular first and second molars. A recent study demonstrated statistically significant better cleaning of apical isthmi when using laser-activated irrigation and the EDDY system compared to ultrasonic irrigation systems [27].

The present results found no significant correlation between the root canal morphology of mandibular teeth with gender similar to the available literature [19, 25]. Also, no significant correlation was found between the prevalence of isthmi in most mandibular teeth with age, except in the mesial root of the second left molar (7LM), where there was a statistically significant difference between the mean age of individuals based on the presence or absence of an isthmus in the root. The mean age of individuals who had an isthmus in the root of the mentioned tooth was lower than the mean age of individuals who did not have an isthmus in the root of the mentioned tooth (*P* value < 0.05). Similarly, Hu et al. [22] showed an inverse correlation between age and prevalence of isthmi in the mesial root of mandibular first molars in a Chinese population. It seems that continued apposition of dentin causes less frequent isthmi in the elderly, emphasizing the higher importance of irrigation in younger patients.

Regarding the root canal morphology, the current results showed that Vertucci's Types I and III were the most common in central and lateral incisors and canine teeth, Types I and V were the most common in first premolars, Type I was the most common in second premolars, Types IV and II were the most common in mesial root of first and second molars, and Type I was the most common in distal root of first and second molars. Other studies conducted in Turkey and China [28, 29] morphologically assessed the mandibular incisor root canal using CBCT and demonstrated that Types I and III were the most common root canal morphologies, which was consistent with the present results. Similarly, Talabani [30] reported that Type I canal configuration is the most prevalent in mandibular anterior teeth in the Iraqi subpopulation.

Arslan et al. [21]. in their study on a Turkish population, evaluated the root canal morphology of mandibular first and second premolars using CBCT images and reported results highly similar to the present findings. The most common root canal morphology of first premolars was Type I (71.4%) followed by Type V (20.1%). Also, Type I was dominant in over 90% of second premolars. The root canal morphology of mandibular premolars was assessed in different parts of Iran, including the cities of Mashhad [31], Qazvin [32], and Isfahan [33]. Their results were in agreement with the present findings. Roy et al. [34] evaluated the root canal morphology of mandibular posterior teeth in an Indian population by CBCT and reported that Vertucci's Type I was the most common (> 95% frequency) in second premolars, which was like the present findings. Also, Type I was the most common in the distal root of second molars in their study, which agreed with the present results, although the obtained frequency values were different (> 90% in the present study versus 14.5 in their study). Consistent with the present results, type IV was the most common morphology in the mesial root of the first and second molars in their study. Gomez, Brea, and Gomez-Sosa [35], in their study, analyzed the root morphology of mandibular second molars and reported that Types II and IV were, respectively, the most common morphological types in the mesial root, while type I was the most common type in the distal root; in our study, Type IV was more prevalent than Type II in mesial roots.

Ahmed et al. [36] proposed a novel classification system for root canal configurations. They argue that their system offers advantages over Vertucci's classification by accommodating a broader range of canal configurations, addressing the issue of “nonclassifiable” canals often encountered with Vertucci's method. The authors designed their system to be more user-friendly and practical for various dental professionals, including clinicians, researchers, educators, and students. However, in this study, Vertucci's classification was employed as it adequately categorized most of the observed configurations. This choice also facilitates comparison with a larger body of existing research that utilizes Vertucci's system.

In the present study, gender had a significant correlation with the root morphology of the left first premolar and right canine. For the left first premolar, Type I morphology was significantly more common in females, while Type V was significantly more common in males. Conversely, for the canine tooth, Type I was significantly more common in males, and Types III and V were significantly more common in females. No other significant correlations were noted. Other studies on this topic found no significant correlation between gender and root canal morphology [21, 28, 29, 34]. Only Iqbal et al. [4] reported that the prevalence of canal variations in mandibular canines was higher in females than in males (*P* = 0.002) in the Saudi population, which agreed with our study.

The present results found no significant correlation between the root canal morphology of mandibular teeth and age, which was in line with the available literature on this topic [28].

Root canal treatment is aimed at eliminating pain, enhancing quality of life, and promoting tissue healing by eradicating the source of endodontic disease [1]. However, this process, particularly the negotiation and preparation of isthmus, may weaken the root structure, especially in teeth like mandibular molars, where the isthmus area is prone to fractures. Research indicates that cleaning and shaping these parts can further reduce fracture resistance, potentially jeopardizing long-term treatment outcomes [37, 38].

The presence of isthmi within root canal systems can harbor debris and promote microbial growth, further complicating treatment success. While addressing the etiology of endodontic disease is crucial, clinicians must balance this with preserving the structural integrity of the tooth [1, 6].

Currently, there is a lack of data regarding the long-term impact of isthmus preparation on nonsurgical endodontic treatment outcomes [9, 12]. To fully understand this relationship and optimize treatment strategies, further research involving long-term patient follow-up is necessary.

## 5. Conclusion

The present results showed that Types I and III were the most common root canal morphology in central and lateral incisors and canines, while Types I and V were the most common in first and second premolar teeth. Types IV and II were the most common in the mesial root of the first and second molars, and Type I was the most common in the distal root of the first and second molars. The mesial root of the first and second molars had the highest prevalence of isthmi. Also, most isthmi were entirely in the middle third in central and lateral incisors, canines, and first premolars, and entirely in the cervical third in the second premolars and mesial root of first and second molars. In the distal root of the first molars, most isthmi initiated from the cervical third and terminated in the middle third while the location of the isthmi was variable in the distal root of the second molars.

## Figures and Tables

**Table 1:** Frequency of root canal morphology of mandibular teeth on the right side in different genders and according to Vertucci's classification.

**Vertucci' class**	**I**	**II**	**III**	**IV**	**V**	**VI**	**VII**	**VIII**	**Other**	**P** **value**
Central incisor										
M	88	2	78	2	7	1	1		1	0.959
F	97	2	81	1	5	0	1			
T	185	4	156	3	12	1	2		1	
Lateral incisor										
M	83	4	88	1	8	1	1			0.844
F	92	2	76	1	5	0	1			
T	175	6	164	2	13	1	2			
Canine										
M	148	1	14	5	1		1			0.923
F	150	2	15	7	0					
T	298	3	29	12	1					
First premolar										
M	52	3	6	1	44				1	< 0.001^a^
F	141	2	6	0	9				1	
T	193	5	12	1	53	2			2	
Second premolar										
M	86	1	1	2	1					0.639
F	94	1	2	0	3	1				
T	180	2	3	2	4	1				
First molar mesial root										
M	1	12	1	22	1					0.976
F	1	13	0	20	1					
T	2	25	1	42	2					
First molar distal root										
M	26	3	1	3	2					0.936
F	24	5	2	4	2					
T	50	8	3	7	4					
Second molar mesial root										
M	9	24	4	35	7				1	0.977
F	10	29	3	37	6				2	
T	19	53	7	72	13				3	
Second molar distal root										
M	73	2	2	1	1				1	0.924
F	80	2	1	2	0				2	
T	153	4	3	3	1				3	

Abbreviations: M, male; F, female; T, total

^a^Statistically significant.

**Table 2:** Frequency of root canal morphology of mandibular teeth on the left side in different genders and according to Vertucci's classification.

**Vertucci's class**	**I**	**II**	**III**	**IV**	**V**	**VI**	**VII**	**VIII**	**Other**	**P** **value**
Central incisor										
M	100	3	82	1	9				1	0.904
F	84	3	70	2	7	1	1			
T	184	6	152	3	16	1	1		1	
Lateral incisor										
M	87	3	91	3	4	1				0.372
F	91	1	79	0	7	1	1			
T	178	4	170	3	11	2	1			
Canine										
M	182	1	11	3	3					< 0.001^a^
F	119	1	22		13					
T	301	2	33		16					
First premolar										
M	106	1	2	0	27	1				0.724
F	101	1	3	2	24	0				
T	207	2	5	2	51	1				
Second premolar										
M	89	2	3		7					0.690
F	85	1	2		3					
T	174	3	5		10					
First molar mesial root										
M	1	11	1	26						0.946
F	1	12	1	22						
T	2	23	2	48						
First molar distal root										
M	21	4	1	4	1					0.928
F	34	5	1	3	1					
T	55	9	2	7	2					
Second molar mesial root										
M	3	31	5	39	6			1	2	0.714
F	5	33	3	43	6			0	0	
T	8	64	8	82	12			1	2	
Second molar distal root										
M	81	2	0	1	0				2	0.068
F	83	2	4	0	2				0	
T	164	4	4	1	2				2	

Abbreviations: M, male; F, female; T, total

^a^Statistically significant.

**Table 3:** Frequency of isthmi in the root canals of mandibular teeth on the right and left sides.

**Side**	**Right**			**Left**		
**Isthmus tooth type**	**Present**	**Absent**	**Total**	**Present**	**Absent**	**Total**
Central incisor	25 (6.9%)	339 (93.1%)	364 (100%)	24 (6.6%)	339 (93.4%)	363 (100%)
Lateral incisor	36 (9.8%)	333 (90.2%)	369 (100%)	50 (13.8%)	313 (86.2%)	363 (100%)
Canine	9 (2.6%)	343 (97.4%)	352 (100%)	10 (2.9%)	332 (97.1%)	342 (100%)
First premolar	3 (1.1%)	265 (98.9%)	268 (100%)	9 (3.4%)	257 (96.6%)	266 (100%)
Second premolar	3 (1.6%)	187 (98.4%)	190 (100%)	3 (1.6%)	189 (98.4%)	192 (100%)
First molar mesial root	46 (60.5%)	30 (39.5%)	76 (100%)	50 (69.4%)	22 (30.6%)	72 (100%)
First molar distal root	15 (20%)	60 (80%)	75 (100%)	15 (20.8%)	57 (79.2%)	72 (100%)
Second molar mesial root	113 (63.8%)	64 (36.2%)	177 (100%)	107 (63.7%)	61 (36.3%)	168 (100%)
Second molar distal root	2 (1.1%)	175 (98.9%)	177 (100%)	3 (1.8%)	164 (98.2%)	167 (100%)

**Table 4:** Prevalence of isthmus and its relation to age (independent sample *T*-test) (right side).

**Tooth type**	**Isthmus**	**No.**	**Age**		**P** **value**
			**Mean**	**S. D**	
Central incisor	Yes	25	40.56	9.942	0.936
	No	325	40.74	11.185	
Lateral incisor	Yes	36	38.53	11.795	0.171
	No	319	41.23	11.13	
Canine	Yes	9	42.78	11.333	0.608
	No	331	40.87	10.995	
First premolar	Yes	3	33.33	2.082	0.319
	No	254	39.46	10.592	
Second premolar	Yes	3	39.67	11.846	0.856
	No	181	38.53	10.752	
First molar-mesial	Yes	46	31.83	12.63	0.19
	No	30	35.4	9.554	
First molar-distal	Yes	15	30.93	8.836	0.372
	No	60	33.95	12.204	
Second molar-mesial	Yes	111	37.05	10.639	0.084
	No	63	40	10.966	
Second molar-distal	Yes	2	41	8.485	0.706
	No	172	38.09	10.86	

*Note:* Alpha level = 0.05.

Abbreviations: S.D, standard deviation.

**Table 5:** Prevalence of isthmus and its relation to age (independent sample *T*-test) (left side).

**Tooth type**	**Isthmus**	**No.**	**Age**		**P** **value**
			**Mean**	**S.D**	
Central incisor	Yes	24	39.21	9.288	0.512
	No	325	40.76	11.286	
Lateral incisor	Yes	49	41.98	12.279	0.344
	No	300	40.36	10.921	
Canine	Yes	10	40.3	12.455	0.996
	No	320	40.28	10.908	
First premolar	Yes	9	42.67	10.909	0.299
	No	248	38.95	10.528	
Second premolar	Yes	3	46.33	6.028	0.18
	No	184	38.41	10.153	
First molar-mesial	Yes	48	34.94	12.288	0.732
	No	22	33.86	11.829	
First molar-distal	Yes	15	31.67	11.223	0.292
	No	55	35.4	12.266	
Second molar-mesial	Yes	104	35.93	9.869	0.014
	No	59	40.12	11.224	
Second molar-distal	Yes	3	31.33	5.508	0.318
	No	159	37.47	10.558	

**Table 6:** Frequency distribution of the location of isthmi in the root canals of right mandibular teeth.

**Location of isthmus** **Tooth type**	**CT-CT**	**CT-MT**	**CT-AT**	**MT-MT**	**MT-AT**	**AT-AT**	**Total**
Central incisor	3 (12%)	1 (4%)	1 (4%)	17 (68%)	0	3 (12%)	25 (100%)
Lateral incisor	5 (13.9%)	1 (2.8%)	1 (2.8%)	26 (72.2%)	1 (2.8%)	2 (5.6%)	36 (100%)
Canine	2 (22.2%)	1 (11.1%)	0	6 (66.7%)	0	0	9 (100%)
First premolar	1 (33.3%)	0	0	2 (66.7%)	0	0	3 (100%)
Second premolar	1 (33.3%)	1 (33.3%)	0	1 (33.3%)	0	0	3 (100%)
First molar mesial root	26 (56.5%)	10 (21.7%)	4 (8.7%)	5 (10.9%)	0	1 (2.2%)	46 (100%)
First molar distal root	3 (20%)	9 (60%)	0	2 (13.3%)	0	1 (6.7%)	15 (100%)
Second molar mesial root	54 (47.8%)	37 (32.7%)	10 (8.8%)	9 (8%)	1 (0.9%)	2 (1.8%)	113 (100%)
Second molar distal root	1 (50%)	0	1 (50%)	0	0	0	2 (100%)

**Table 7:** Frequency distribution of the location of isthmi in the root canals of left mandibular teeth.

**Location of isthmus** **Tooth type**	**CT-CT**	**CT-MT**	**CT-AT**	**MT-MT**	**MT-AT**	**AT-AT**	**Total**
Central incisor	3 (12.5%)	0	1 (4.2%)	17 (70.8%)	0	3 (12.5%)	24 (100%)
Lateral incisor	4 (8%)	3 (6%)	0	40 (80%)	0	3 (6%)	50 (100%)
Canine	1 (10%)	0	0	9 (90%)	0	0	10 (100%)
First premolar	2 (22.2%)	1 (11.1%)	0	5 (55.6%)	0	1 (11.1%)	9 (100%)
Second premolar	2 (66.7%)	0	0	1 (33.3%)	0	0	3 (100%)
First molar mesial root	26 (52%)	13 (26%)	5 (10%)	5 (10%)	0	1 (2%)	50 (100%)
First molar distal root	5 (33.3%)	7 (46.7%)	2 (13.3%)	1 (6.7%)	0	0	15 (100%)
Second molar mesial root	55 (51.4%)	35 (32.7%)	9 (8.4%)	7 (6.5%)	0	1 (0.9%)	107 (100%)
Second molar distal root	0	2 (66.7%)	0	1 (33.3%)	0	0	3 (100%)

## Data Availability

The data used to support the findings of this study were supplied by the corresponding author under license and data will be available on request up to 1 year from publication date. Requests for access to these data should be made to the corresponding author.
